# Characterization of Asian Corn Borer Resistance to Bt Toxin Cry1Ie

**DOI:** 10.3390/toxins9060186

**Published:** 2017-06-07

**Authors:** Yueqin Wang, Jing Yang, Yudong Quan, Zhenying Wang, Wanzhi Cai, Kanglai He

**Affiliations:** 1 State Key Laboratory for Biology of Plant Diseases and Insect Pests, Institute of Plant Protection, Chinese Academy of Agricultural Sciences, Beijing 100193, China; yueqinqueen@126.com (Y.W.); cutejingyang@163.com (J.Y.); yudongquan1@126.com (Y.Q.); wangzy61@163.com (Z.W.); 2 Department of Entomology, China Agricultural University, Beijing 100193, China; caiwz@cau.edu.cn

**Keywords:** *Ostrinia furnacalis*, *Bacillus thuringiensis*, inheritance, cross-resistance, resistance management

## Abstract

A strain of the Asian corn borer (ACB), *Ostrinia furnacalis* (Guenée), has evolved >800-fold resistance to Cry1Ie (ACB-IeR) after 49 generations of selection. The inheritance pattern of resistance to Cry1Ie in ACB-IeR strain and its cross-resistance to other Bt toxins were determined through bioassay by exposing neonates from genetic-crosses to toxins incorporated into the diet. The response of progenies from reciprocal F_1_ crosses were similar (LC_50_s: 76.07 vs. 74.32 μg/g), which suggested the resistance was autosomal. The effective dominance (*h*) decreased as concentration of Cry1Ie increased. *h* was nearly recessive or incompletely recessive on Cry1Ie maize leaf tissue (*h* = 0.02), but nearly dominant or incompletely dominant (*h* = 0.98) on Cry1Ie maize silk. Bioassay of the backcross suggested that the resistance was controlled by more than one locus. In addition, the resistant strain did not perform cross-resistance to Cry1Ab (0.8-fold), Cry1Ac (0.8-fold), Cry1F (0.9-fold), and Cry1Ah (1.0-fold). The present study not only offers the manifestation for resistance management, but also recommends that Cry1Ie will be an appropriate candidate for expression with Cry1Ab, Cry1Ac, Cry1F, or Cry1Ah for the development of Bt maize.

## 1. Introduction

The Asian corn borer (ACB), *Ostrinia furnacalis* (Guenée) (Lepidoptera: Crambidae), is the most destructive insect pest of maize throughout the China. Bt maize hybrids expressing Cry1Ab toxin (containing events MON810 (Monsanto) and Bt11 (Syngenta)), as well as Cry1Ie-expressing maize developed by the Institute of Crop Sciences, Chinese Academy of Agricultural Sciences, can provide excellent protection from the ACB and cotton bollworm, *Helicoverpa armigera* [1,2,3]. However, a widespread and prolonged use of Bt crops could rapidly lead to the evolution of resistance within target pest populations [4,5,6,7]. Continuous exposure of Bt has displayed a great potential of resistance in numerous lepodopteran pests including cotton bollworm [8], the ACB [9,10], and the European corn borer, *Ostrinia nubilalis* [11], in the laboratory selection experiments.

In order to achieve the sustainable utilization of this technology, it is necessary to adopt appropriate resistance management strategies. Although several strategies have been proposed to manage target insects resistance to Bt maize, the high-dose/refuge approach and multi-gene strategy (pyramiding two or more toxins with different modes of action) have been most recommended [12,13]. One of two critical assumptions of the high-dose/refuge strategy documented to diminish evolution of resistance in target insects for Bt crops expressing Bt toxins is recessive or incompletely recessive inheritance of resistance, i.e., progenies from mating between homozygous susceptible and homozygous resistant adults are susceptible to the high-dose expression [14]. To some extent, the fitness of heterozygous individuals is lower on a Bt crop than the homozygous resistant parent, the maximum interruption in resistance can be acquired with resistance traits that are functionally recessive [15,16].

Pyramiding of two or more toxins with different binding receptor molecules and different modes of action in the larval midgut is effective [13,17]. Multiple mutations for resistance to both toxins are required simultaneously under the circumstances, the homozygous resistance individuals arising would be extremely rare. In case of any resistance to Bt toxins remaining stable while selection is stopped, pyramiding toxins is superior to that of a rotation of different toxins. The ability of applying toxin mixtures or rotations of different toxins is greatly enhanced if the resistance allele to each toxin is recessive [18].

It is essential to understand the characteristic of resistance to Bt toxins for evaluating the risk of resistance and implementing strategies to establish an effective IRM program. Our primary objective here was to estimate the pattern of inheritance of resistance to Cry1Ie in a laboratory-selected ACB strain ACB-IeR. Possible sex linkage/maternal effects and dominance were determined through performing Mendelian cross assays, while backcrossing experiments were performed to estimate the number of loci influencing the resistance. In addition, Bt maize (expressing Cry1Ie toxin) plant tissue bioassays were carried out to measure the fitness of susceptible and resistant insects as well as the F_1_ progeny. Besides, cross-resistance patterns to those Bt toxins expressed in most commercialized Bt maize and Bt cotton events such as MON810, Bt11, TC1507, and MON532 were also assessed in ACB-IeR strain. The results of this research would provide valuable information for initiating an effective IRM program for prospective Cry1Ie-containing maize adopted in China.

## 2. Results

### 2.1. Evolution of Resistance

After having been selected for 49 generations with increased Cry1Ie concentration during subsequent generations, a Cry1Ie-resistant strain (ACB-IeR) was established through laboratory selection experiments using artificial diet mixed with Cry1Ie toxin. Based on bioassays for evaluating the susceptibility to Cry1Ie toxin in ACB-BtS and ACB-IeR strains, the LC_50_ value was significantly higher in ACB-IeR strain compared with ACB-BtS, which showed a resistance ratio of more than 800-fold and demonstrated that resistance to Cry1Ie toxin was achievable for this insect (Table 1).

### 2.2. Cross Resistance

The LC_50_ values for Cry1Ab, Cry1Ac, Cry1Ah, and Cry1F toxins were not significantly different in ACB-IeR strain compared to the ACB-BtS strain (Table 1), indicating that the ACB-IeR strain is not cross-resistant to these four Bt toxins.

### 2.3. Maternal Effects and Sex Linkage

F_1_ offspring of reciprocal crosses tested with Cry1Ie were intermediate in resistance to their respective susceptible and resistant parents, with LC_50_ values of 76.07 μg/g and 74.32 μg/g, which were greater than the LC_50_ of the susceptible parental strain (1.10 μg/g) and significantly less than the LC_50_ of the resistant parental strain (more than 940 μg/g) (Table 2). The LC_50_ values of the F_1_ offspring were not significantly different from one another (RR (95% CI) = 1.02 (0.76–1.37)), indicating that inheritance was autosomal, with no sex link.

### 2.4. Number of Loci Influencing Resistance

The fitness test for goodness-of-fit to a monofactorial model showed that backcross progeny had higher actual mortality than expected mortality resulting from all of the five Cry1Ie toxin doses in a series, i.e., the pattern of response was not consistent with a monofactorial model (∑χ^2^ = 62.02 > ∑χ^2^_0.05_ = 3.84, df = 1) (Table 3). The null hypothesis is rejected, which indicates that the inheritance of the resistance to Cry1Ie in ACB-IeR strain may be under polygenic control.

### 2.5. Dominance

The effective dominance level, *h*, based on toxin diet bioassay, varied widely with the Cry1Ie toxin concentration, from incompletely dominant inheritance at low concentrations to incompletely recessive inheritance at high concentration (Table 4). For example, the resistance was incompletely dominant at the concentration of 0.5 μg/g (*h* = 0.96), and declined to incompletely recessive at 100 μg/g (*h* = 0.35).

*h* values based on the plant tissues bioassays were 0.02 and 0.98 for Cry1Ie maize leaves and silks (Table 5), which indicated that the resistance was functionally recessive at the whorl stage of maize plant development and more dominant at the silk stage of maize plant development.

## 3. Discussion

Our efforts to select for resistance to Cry1Ie in the ACB have resulted in a high level of resistance (more than 800-fold) after selecting for 49 generations, among those reported for the ACB strains selected with Cry1Ab (39.4-fold) and Cry1Ac (78.8-fold) [10]. This result indicates that the population may have genetic variation for resistance and would be expected to attain higher levels of resistance if exposure to Cry1Ie continued. In the lab, factors contributing to the differences in resistance levels may include selection pressure, toxic materials tested (protoxin and trypsinized toxin), the number of generations selected, differences in proteolytic activation, detoxification, and even the susceptibility among the unselected strains used for calculating resistance ratios [19,20,21]. However, for the insect populations that are resistant to Bt crops from the field, the initial frequency of resistance alleles, the scale of refuge populations, the toxin-expression level of Bt crops during different plant stage, and effective dominance will strongly affect the rate of resistance evolution in a population [16,22]. The time exposure to Bt crops, resistance genes, and the genetic background of different populations may also have contributed to its increased tolerance to Bt crops [23].

Resistance to Cry1Ie in ACB-IeR strain was not sex linkage and not maternal influenced. These results are consistent with nearly all previous results with Bt resistance, including resistance to Cry1Ab in *Mythimna unipuncta* [24], the ACB to Cry1Ab and Cry1Ac [10], *H. armigera* to Cry1Ac [25], *O. nubilalis* to Cry1Ab [26], greenhouse-derived strain of *Trichoplusia ni* to Cry1Ac, and *B. thuringiensis* subsp. *Kurstaki* [27,28]. Many studies suggest that autosomal inheritance of Cry resistance may be common in a number of lepidopterous insects. However, resistance to Cry1Ac in Malaysian populations of *Plutella xullostella* had some maternal influence [29]. Also, inheritance patterns of resistance to Cry3Bb1 in *Diabrotica virgifera virgifera* is sex-linkage [30]. These significant differences demonstrate that it is vital demand for understanding species-specific genetic model of resistance to program case specific resistance management strategy.

Genetic-crosses showed that the effective dominance level (*h*) of Cry1Ie resistance depended on the concentration of the toxin, with resistance more dominant as concentration decreased. Increased dominance of Bt resistance at low toxin concentrations has been reported in several other species [6,25,26]. Many factors contribute to dominance. In a related example, the dominance of resistance to Bt toxins depended on the toxins, resistance levels, and the particular strains [20]. Greenhouse tests indicated that dominance may vary depending on different levels of Bt toxin expression in tissues during different plant stages (e.g., vegetative-stage and reproductive-stage) [6]. This was also found in the present study, i.e., the Cry1Ie-resistance was nearly completely recessive on Cry1Ie maize leaf, but nearly completely dominant on Cry1Ie maize silk. This suggests that a high dose of Cry1Ie in transgenic maize is necessary for a refuge strategy to be successful in delaying resistance. The estimates of dominance are based on the assumption that the parent populations were completely homozygous when F_1_ progeny were produced. The presence of heterozygotes in resistant strain would tend to lower the survival rate of F_1_ progeny and thus underestimate the degree of dominance. In field, the more the effective dominance of inheritance increases, the more quickly it is able to develop resistance [31].

The indirect tests employed to estimate the number of genes contributed to resistance on the basis of the actual mortality and expected mortality of backcross progeny indicated that more than one locus involved in resistance in ACB-IeR. The polygenic nature of resistance attributed to an increase in resistance to Cry1Ie with continuous and additional selection. If the resistance results from one locus with two alleles, the sole RR allele would have been fixed within several generations of selection and further increase in resistance would not have happened [32]. 

The genetic basis for resistance to Bt toxins appear to species- and/or strain-specific under different selection regimes (weaker selection can allow polygenic weak resistance mechanisms to develop through multiple small increases in fitness). For instance, resistance to Cry1Ab is characterized as polygenic and monogenic in Cry1Ab- and Cry1Ac-selected strains of the ACB, respectively. In contrast, resistance to Cry1Ac is characterized as primarily monogenic in both Cry1Ab- and Cry1Ac-selected strains [10]. In field-derived populations of the diamondback moth, resistance to Cry1Ac is controlled by more than one allele on separate loci in a population from Malaysia [33], and resistance to Cry1Ab is conferred by two difference genes in a population from the Philippines [34]. Besides, the number of loci engaged in resistance may vary for the selection with different compositions of the toxins [35].

The development of resistance to one toxin can lead to cross-resistance to other Bt toxins [36,37]. Selection for Cry1Ie resistance resulted in no cross-resistance to Cry1Ab and Cry1Ac, and did not reduce the susceptibility of the ACB to Cry1F and Cry1Ah. This result is consistent with previous study [38]. Binding to different receptors on brush border membrane vesicles (BBMV) may account for the absence of cross-resistance among those proteins. To prove this point, receptor binding studies and identification of the Cry receptors in *O. furnacalis* is needed. Evidence for Cry1Ia7 sharing binding sites with Cry1Ab or Cry1Ac toxins have not been detected in *Earias insulana* and *Lobesia botrana* [39]. The level of cross-resistance is closely related to the Bt toxins molecular structure. For example, a high level of cross-resistance among Cry1Ab and Cry1Ac is seriously intended as there are 85% similarities in their amino acid sequence [40,41]. Evidence for successful to against the ACB as well as Cry1Ac-resistant *H. armigera* have been revealed in transformed plants expressing Cry1Ie toxin [3,42], and the absence of cross-resistance between Cry1Ie and other toxins suggests that maize hybrids with these pyramided toxins would offer viable alternative combination to implement strategy for resistance management. This is in accordance with the fundamental assumption that a species little ability evolve resistance to both toxins simultaneously with independent mutations in the genes encoding the receptors.

## 4. Conclusions

The present study demonstrates that a laboratory-selected ACB strain has evolved a significant level of resistance to Cry1Ie toxin, but without cross-resistance to other Bt toxins such as Cry1Ab, Cry1Ac, Cry1Ah, and Cry1F. The genetic model of resistance to Cry1Ie in ACB-IeR strain is polyfactorial. *h* was recessive inheritance at Cry1Ie maize whorl leaves assays and incomplete dominant inheritance at silks assays. The results also suggest that pyramiding of Cry1Ie and Cry1Ab or Cry1Ac, Cry1Ah, or Cry1F can be used as an IRM tactic for improving resistance management strategies.

## 5. Materials and Methods

### 5.1. The ACB Strains and Genetic Crossess

Two strains of the ACB, a Bt susceptible strain (ACB-BtS) and a Cry1Ie-resistant strain (ACB-IeR) were colonized in the laboratory. The ACB-BtS strain, originating from a field collection in Liaoning Province within the corn region of northeastern China, had been reared using standard rearing techniques for 23 generations without exposure to any insecticide before bioassays were conducted [43].

The ACB-IeR strain was selected from a laboratory colony derived from a field collection (88 pairs of female and male moths derived from 948 diapause larvae) in Shaanxi Province in 2010 (Bt spraying is hardly practiced in this area. In addition, Bt maize has not been commercialized in China). Then selection regime was exposing larvae to an artificial diet incorporated with Cry1Ie toxin, of which the toxin concentration was initially at 50 ng/g (toxin/diet) and steadily increased generation by generation up to 6.4 μg/g in the 14^th^ generation, which offered a successful attempt to maintain the intensity of selection. Thereafter, the selecting pressure had been maintained at 6.4 μg/g in the next 35 generations. Briefly, genetic model of resistance to Cry1Ie was investigated of the 49^th^ generation.

Larvae were incubated at 27 ± 1 °C with a 16:8-h light:dark (L:D) photoperiod and 80% relative humidity (RH) on an agar-free semiartificial diet [43]. Pupae were transferred to mating cages with more than 80% RH and a photoperiod of 16:8 h (L:D). A piece of waxed paper as an egg depositing substrate was placed on the top of the cage and collected daily. Egg masses were incubated in plastic boxes lined with moistened filter paper until hatching.

Before eclosion, the sex of pupae was distinguished visually and isolated for either ACB-BtS or ACB-IeR [44], then the reciprocal crosses were made between two strains. Eggs derived from those adults were incubated in the insectary to provide neonates for subsequent bioassays and/or for rearing to adults to produce backcross population through mating with ACB-IeR strain.

### 5.2. Bt Toxins

Cry1Ab and Cry1F toxins (98% pure protein), used for bioassays were Trypsin-activated and produced by Marianne P. Carey, Case Western Reserve University, USA. Trypsin-activated and chromatogrsphically purified Cry1Ac toxin was brought from Envirologix. Chromatographically purified Cry1Ie, a recombinant protein, was extracted from *E. coli*. Cry1Ah toxin was expressed in the *Bacillus thuringiensis* acrystalliferous mutant HD73^−^. Purity of both toxins are >85% pure protein.

### 5.3. Plant Materials

Bt transgenic maize (event IE034) expressing Cry1Ie toxin and its negative counterpart control were grown at the experimental farm of China Agricultural University, Beijing. There was no application of pesticides for the entire corn growing season.

### 5.4. Toxin Diet Bioassays

The susceptibility of neonates to Bt toxins was determined in survival bioassays by exposing neonates (<12 h after hatching) to serially diluted Bt toxins incorporated into the agar-free semi-artificial diet [45]. A single neonate was randomly transferred into each well of 48-well tray and then covered with a piece of paper and the lid. Trays were held in a growth chamber for seven days at 27 °C and 80% RH under a 16 h photophase. Survivor number and the weight of larvae surviving per tray were recorded after seven days of exposure. If a larva had not developed beyond the first instar and weighed ≤0.1 mg, it would be counted as dead for calculating practical mortality. Average larval weight of survivors would be used to determine the larval growth inhibition rate as a function of toxin concentration. Bioassays were repeated on two dates with total of 96 larvae per concentration and included 6–10 concentrations of purified toxin. Dilutions of toxins were prepared in distilled water. Distilled water was used as a control.

Toxicities of with Cry1Ab, Cry1AC, Cry1Ah, and Cry1F toxins were bioassayed using the methods described above. LC_50_ values were used to estimate the cross-resistance to those toxins in ACB-IeR.

### 5.5. Plant Tissue Bioassays

The whorl leaves sampled from the plant at V5–V8 stages were subjected to bioasssy. A piece (about 1 cm × 1 cm) of leaf was placed in each individual well of a 24-well rearing tray, which was infested with a neonate larva per well, and then lidded with a piece of moistened filter paper lining the top of the tray. There were 96 larvae assayed for each treatment. The bioassay experiments were replicated two times.

Bundles of fresh silks were sampled from different Bt maize and non-Bt maize plants at silking stage in the field. Each bundle of silks was placed into a plastic container lined with moistened filter paper. Each container was then infested with 10 neonates (<12 h after hatching) using a fine brush. In each bioassay treatment, there were four containers per treatment. The experiment was repeated twice on two different dates.

All rearing trays and containers were kept in an incubator at 27 °C and 80% RH under an 8 h scotophase. The number of surviving larvae or weights were recorded either daily or every two days, and fresh tissue was provided when necessary.

The traits used in the calculation of dominance were larval survival seven days after infestation.

### 5.6. Statistical Analysis

#### 5.6.1. Evolution of Resistance

Probit regression lines were calculated based on concentration response data of each strain and/or genetic cross to Bt toxins using PoloPlus (LeOra Software), which would generate median lethal concentrations (LC_50_) values with 95% fiducial limits (FL), Chi-Squared (χ^2^), slope with standard errors (Slope ± SE), and resistance ratio (RR) with 95% confidence intervals. RR is calculated based on the LC_50_ values for the resistant strain or progeny from a cross relative to a susceptible strain. If a 95% confidence interval includes 1, then the LC_50_s are not significantly different [46].

#### 5.6.2. Sex Linkage Analysis

Maternal effects or sex linkage on ACB-IeR strain were examined by comparing LC_50_s of the two F_1_ reciprocal crosses between ACB-IeR and ACB-BtS strains. If there is no significant difference in LC_50_ values of the two F_1_ reciprocal crosses, then inheritance of resistance is regarded as autosomal. Conversely, if LC_50_ values of the two F_1_ reciprocal crosses are significantly different, inheritance of resistance is regarded as sex linkage.

#### 5.6.3. Dominance of Resistance Test

The single-concentration method to estimate effective dominance (*h*) was used for analysis of dominance.

*h* = (W_12_ − W_22_)/(W_11_ − W_22_) (1)

W_11_, presumed to be 1 at all treatment concentrations, is the fitness of resistant parent; W_12_ and W_22_ are the fitness of F_1_ progenies and susceptible parent, which were estimated as ratio of the survival rate of F_1_ progenies and/or susceptible larvae to the survival rate of the resistant parent at a specific treatment concentration, respectively [47,48]. *h* value ranges from 0 (completely recessive) to 1 (completely dominant), with 0.5 indicating co-dominance or additive inheritance. Therefore, 0 < *h* < 0.5 and 0.5 < *h* < 1 are defined as incompletely recessive and incompletely dominance, respectively.

#### 5.6.4. Number of Loci Influencing the Inheritance Test

Test for fitting the monogenic model of resistance was evaluated through assessing the corresponding chi-square (χ^2^) values. The observed and expected mortalities of the backcross population at different Cry1Ie concentrations were evaluated with χ^2^-test for fitting the Mendelian single gene model of resistance [49,50]. If the resistance is controlled by one locus with two alleles, the backcross of F_1_ (RR × SS) × RR will produce 50% RS and 50% RR offspring. Mortality probabilities estimated at concentration *i* for assumed F_1_ offspring (*M_RS_*) and resistant parent (*M_RR_*) genotypes were used to estimate the expected mortality *p_i_* in the backcross progeny at toxin dose *i* [49] as

(2) $$p_{i}=0.5\left(M_{RS}+M_{RR}\right)$$

The difference between the observed and expected number of deaths in the backcrosses were analyzed with χ^2^-test for goodness-of-fit as

(3) $$\chi^{2}=\sum {\left(f_{i}-np_{i}\right)}^{2}/npq$$

where $f_{i}$ is the number of observed dead larvae in backcross survival bioassays at dose *i*, *n* is the number of larvae exposed to dose *i*, and *q_i_* = 1 − *p*. Then the sum of χ^2^ (∑χ^2^) at each concentration was compared with a χ^2^ distribution with one degree of freedom. The inheritance of resistance is expected to fit the monofactorial model if ∑χ^2^ < χ^2^_0.05_ (df = 1) [49].

## Figures and Tables

**Table 1:** Susceptibility of the Asian corn borer (ACB-BtS, ACB-IeR) to 5 Bt toxins

Bt Toxin	Strain	*n* ^a^	LC_50_ (95% FL) μg/g	RR ^b^ (95% CI)	Slope ± SE	χ^2^	df (χ^2^)
Cry1Ie	ACB-BtS	576	1.10 (0.86–1.28)	1	7.31 ± 1.29	14.2	10
	ACB-IeR	864	>940	>854.5	nd	nd	16
Cry1Ab	ACB-BtS	576	0.21 (0.14–0.30)	1	2.16 ± 0.47	7.7	10
	ACB-IeR	672	0.17 (0.09–0.25)	0.8 (0.5–1.2)	1.46 ± 0.19	11.7	12
Cry1Ac	ACB-BtS	576	0.27 (0.19–0.34)	1	1.70 ± 0.26	7.1	10
	ACB-IeR	576	0.21 (0.15–0.30)	0.8 (0.5–1.3)	1.31 ± 0.20	6.6	10
Cry1Ah	ACB-BtS	576	0.20 (0.09–0.28)	1	1.98 ± 0.53	7.9	10
	ACB-IeR	576	0.20 (0.07–0.30)	1.0 (0.6–1.6)	1.81 ± 0.35	12.7	10
Cry1F	ACB-BtS	576	0.64 (0.42–1.01)	1	2.45 ± 0.45	10.8	10
	ACB-IeR	576	0.59 (0.35–0.86)	0.9 (0.6–1.4)	1.72 ± 0.27	9.2	10

^a^ *n*, number of larvae tested. ^b^ RR, resistance ratio with their 95% confidence intervals compared with susceptible strain at LC_50_. nd, not determined, indicates that the Probit regression line could not be determined because the range of Cry1Ie concentrations needed to cause a significant response exceeded the range tested.

**Table 2:** Responses of F_1_ progenies from reciprocal crosses between resistant and susceptible strains of the Asian corn borer to Cry1Ie toxin.

Cross	*n*	LC_50_ (95% FL) μg/g	RR (95% CI)	Slope ± SE	χ^2^	df (χ^2^)
R_♀_ × S_♂_	672	76.07 (58.85–100.55)	69.2 (56.2–85.1)	2.93 ± 0.63	9.5	12
S_♀_ × R_♂_	768	74.32 (59.37–97.72)	67.6 (52.4–87.1)	2.09 ± 0.43	6.9	14

**Table 3:** Fitness test of monogenic mode to Cry1Ie in Cry1Ie-selected Asian corn borer.

Dosage μg/g	Actual Mortality (%)	Expected Mortality (%)	χ^2^
5	12.5	11.0	0.24
10	18.8	16.7	0.31
50	22.9	22.4	0.01
100	55.2	43.2	5.64
200	96.9	59.4	55.9
∑χ^2^	-	-	62.02

**Table 4:** Effective dominance (*h*) of resistance to Cry1Ie toxin in the Asian corn borer larvae.

Concentration μg/g	Strains	Survival (%)	Fitness *	*h*
0.5	ACB-BtS	72.9	0.76	
	ACB-IeR	95.8	1	
	ACB-IeRS	94.8	0.99	0.96
5	ACB-BtS	0	0	
	ACB-IeR	92.7	1	
	ACB-IeRS	85.4	0.92	0.92
50	ACB-BtS	0	0	
	ACB-IeR	86.5	1	
	ACB-IeRS	68.8	0.79	0.79
100	ACB-BtS	0	0	
	ACB-IeR	84.4	1	
	ACB-IeRS	29.2	0.35	0.35

* Fitness of the susceptible parent and the reciprocal cross was estimated from the survival rate of the larvae at a specific treatment concentration divided by the survival rate of the resistant parent at the same concentration.

**Table 5:** Effective dominance values (*h*) of Cry1Ie resistance in ACB-IeR strain of the Asian corn borer based on Cry1Ie maize plant tissues bioassays.

Plant Tissue	Strains	Survival %	Fitness	*h*
whorl leaves	ACB-BtS	29.2	0.36	
	ACB-IeR	81.8	1	
	ACB-IeRS	30.2	0.37	0.02
silk	ACB-BtS	50.0	0.55	
	ACB-IeR	91.2	1	
	ACB-IeRS	90.0	0.99	0.98
